# Operative vs. Mechanical Dentistry

**Published:** 1875-04

**Authors:** C. E. Case


### OPERATIVE *VS.* MECHANICAL DENTISTRY.

BY C. E. CASE, D. D. S.

It has been suggested by some parties that the separation of these two branches of the profession would be to the interest of both. Without any apologies for my inability to cope with some of the advocates of the movement, I will simply state my views in regard to it. Let us, for the sake of illustration, suppose that the divorce has been effected. What is the result? In the first place, we find a growing indifference on the part of many of our best operators for their brethren of the more lowly, but possibly equally important branch. They will finally come to the conclusion that it is beneath the dignity of the operative dentist to recognize the mere mechanic. On the other hand it will doubtless give rise to a number of dental carpenter shops the object of which will be to supply the people at large with arrangements with which to masticate. They will not be what they should be, unless a law be passed which will demand of the mechanical dentist what the present law does of all who are in the profession in this state. It will not be admitted by any one that any person who has not studied for the profession will be able to make a set of teeth combining the requisite qualities. Or it may be the desire of the advocates of the movement to clear themselvs of all the responsibility, so far as artificial dentures are concerned, in order that they may give their undivided attention to operative dentistry. It will be found then that, although they make more money, the people at large have poorer dentures. It may be also that the intention of some is to have some of the mechanical members of the profession establish laboratories and after the impressions and articulation is taken by them (the operative dentists) have the mechanics make the teeth and of course the credit would belong to the operator. Here now are several points which I dont think any dentist who is an honest gentleman would attempt to controvert. There is already a growing indifference between many of our best operators and the workmen in the laboratory. How often do we hear them say, "Oh! I never have any thing to do with the laboratory work. Indeed I have not done anything in the laboratory for ever so long. My man does all that and I have other things to attend to." I may mistake them, but I always feel as if there was a manifest indifference to the man and his work.

The gentleman in the parlor don't stoop so low as to recognize the man as his equal in skill. This is not an honest way of doing. There is many a prosperous dentist in the land who owes not a little of his reputation to the skill of his man. It is not beneath the dignity of any honest upright dentist to recognize the skill of his assistant or to visit the laboratory once in a while and thus show at least that he thinks there is such a person behind the scenes who wields the hammer, blows the forge, and does the dirty work. If the profession should kick the mechanical dentist out of doors people will still want artificial teeth and the gentleman of the operative persuasion who don't bother with such work refusing to make it, there will be a demand upon the mechanical dentists for it, and it must be admitted that until a man is a pretty fair operator he can not meet all the requirements of the mechanical department. This, then, unless the law I spoke of is brought to bear, will throw open the doors of mechanical dentistry to a large number of professional quacks who will glut the market with things made of rubber and dignified by the name of teeth. It takes years of unremittent study to become a tolerably good dentist. Even then it taxes the ingenuity of a man who all these years has been familiarizing himself with the mouth and teeth to make a set of teeth for all the cases presented. How then shall we expect the mere mechanic to meet the want which the skilled operator sometimes fails upon.

If the responsibility is to be evaded by the operative and thrown on the mechanical dentist, who shall a confiding public rely upon? The operator will say, I fill and save teeth, but do not make them. The mechanic will say, I only make teeth; I did not prepare the mouth; I have not the liberty of treating your mouth as I should desire in order to make a success of the case, The law will not allow me to do anything but make your teeth. Or if the law is in force requiring the mechanical dentist to be a graduate or pass an examination it will be found that as soon as a person graduates or passes the examination he will at once step up a little higher and "Don't make artificial dentures."

As I have said before, the seperation of the two branches may put money in to the pockets of the operators in some places but it certainly will militate against them in others. This will cause a division in the ranks and then possibly ill feelings will prevail between the dentists who are both operators and mechanics and those who are operators only. This will be deplorable. Suppose the laboratories spoken of be started and the operative dentist takes the impression and articulation and the teeth are made by the mechanic at the laboratory. Either the operator must admit that the work is not done in his office and under his supervision, or else he must sail under false colors and claim to have manufactured that which he has had nothing to do with. There are two temptations offered here. If the piece of work is not successful he naturally shifts the responsibility on the maker, and the patient, in disgust, thinks that the operator should have had it made right as he took the impression and bite. Let us suppose however that the piece is a perfect success. How strong the temptation to claim all the glory when none of it of right belongs to him. Is this honesty?

These are only a few of the points which may be successfully used in the argument. Few men are perfect. Few but have their failings. Let us guard against doing anything which will create discord in the ranks of a profession which hitherto has always presented a solid unbroken line of defense to any and all enemies which have threatened an attack. The movement in my judgement will not only create enemies but will put arms into their hands which will certainly be used against us. Anything which is perfection must contain within itself all those elements and accomplishments which are neccessary to perfection and success. Strip it of any one of these elements and what does it become, an absurdity. A failure at which any enemy will point with disdain. No! Gentlemen, let us keep all that we have and attain all that is possible. Our profession has recently made long strides. Why? Because its numbers are increasing and each increase brings more to our help and strength and we will find that "In unity there is strength" In strength there is victory.

